# Assessing Community Empowerment for an Innovative Epidemiologic Approach

**DOI:** 10.3390/healthcare8020173

**Published:** 2020-06-15

**Authors:** Pedro Melo, João Neves-Amado, Alexandra Pereira, Cândida Maciel, Tiago Vieira Pinto, Teresa Cardoso

**Affiliations:** 1 Institute of Health Sciences/Centre for Interdisciplinary Research in Health and Portugal, Universidade Católica Portuguesa, 4169-005 Porto, Portugal; jamado@porto.ucp.pt; 2 Administração Regional de Saúde do Norte, 4000-477 Porto, Portugal; alemnap@gmail.com (A.P.); cmfmaciel@arsnorte.min-saude.pt (C.M.); tpinto@arsnorte.min-saude.pt (T.V.P.); 3 Unidade Local de Saúde de Matosinhos, Public Health Unit, 4464-513 Matosinhos, Portugal; teresa.cardoso@ulsm.min-saude.pt

**Keywords:** public health nursing, community health nursing, community empowerment epidemiological surveillance, nursing diagnoses

## Abstract

Community empowerment can be a process, but also the result of nursing care. To analyze it as a result there is an instrument that allows to quantify its level in nine domains. According to Melo (2020), health centers can be considered communities, becoming the potential target of community and public health nurses care, especially in the public health unit. One of the main functions of a public health unit is the epidemiological surveillance of the population’s health state. However, traditional epidemiological surveillance is focused on diseases and Melo (2020) proposes a new approach for epidemiology focused on people in what concerns nursing diagnosis. The aim of this research is to identify the level of empowerment of four Portuguese primary healthcare structures, named as ACeS, so as to improve the epidemiological surveillance of nursing diagnoses. As methodology, we developed four focus group with all nursing leaders from all primary care units of the four ACeS, using the Portuguese version of the empowerment assessment rating scale. The results present the level of community empowerment of each ACeS according to the nine domains of the scale. The needs of intervention to improve the ACeS empowerment were also identified in order to develop the epidemiological surveillance of nursing diagnoses.

## 1. Introduction

**Figure 1:**
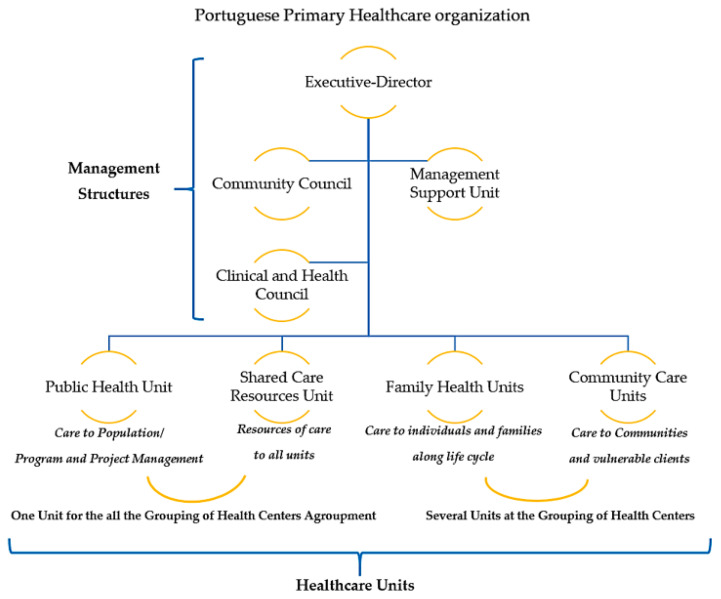
Portuguese primary healthcare organization.

The organization of primary health care in Portugal has its current configuration since 2008 [1]. This configuration presents a structure with a hierarchical organization reporting to the management and a group of different units related do healthcare to individuals, families, communities and population. Together, these management structures and healthcare delivery structures are called “Health Center groups” (ACeS), as represented in Figure 1.

The management of the ACeS has on the top of the hierarchy the “executive-director” (ED). A “community council” (usually led by the local city hall) and a “Management Support Unit” (that leads the financial and resources management) support the ED. The clinical governance related to health care providence (for example the management of specific clinical areas) is provided by a “clinical and health council” composed by a family health physician, that usually coordinates, a community health nurse a public health physician and another health technician that can be, for example, a social worker, a nutritionist or a psychologist. This “clinical and health council” gives support to the ED. The nurse that integrates it has the role of president of the nursing dean of the ACeS.

Regarding healthcare, each ACeS has different types of units:

- “Family health unit” that provides care to individuals and families throughout their life cycle. These units have teams composed by family health physicians, family health nurses and clinical secretaries;
- “Community care unit” provides care to communities (like schools) and to more vulnerable individuals and groups that need specialized care (like mental health care, maternal care, rehabilitation, chronic diseases complex care, etc.). This unit, coordinated by a nurse, has all kinds of specialist nurses and the cooperation of the professionals of the “shared care resources unit” described next;
- “Shared care resources unit” provides specialized care and consulting to clients referenced by all units (like psychology, social care or nutrition);
- “Public health unit” focuses on population care and on the management of health programs and projects; This unit has a multidisciplinary team related to public health;
- Some ACeS also have other kinds of units specialized in care like pneumological diagnostic centers (e.g., diagnose and treatment of tuberculosis) or HIV testing and counselling centers. Figure 1 illustrates the Portuguese primary care organization.

Concerning the public health unit, one of its major roles is to provide the epidemiological surveillance of the population health status. This diagnosis allows the management of the programs and projects of the ACeS [2]. All the primary care units of the ACeS develop these programs responding to the major needs of the population and having the main monitoring provided by the public health unit.

One of the professionals that integrate the public health unit is the community and public health nursing specialist, whose skills are described in the Portuguese law [3]. One of the main skills is to cooperate in the epidemiological surveillance of geodemographic nature. In this matter, the information technology (IT) available in Portuguese health services allow the extraction of data to analyze the nursing records from an epidemiological approach. Portuguese nurses use international classification of nursing practice (ICNP) [4] to record the diagnostic activities, the nursing diagnostic and the interventions. There is a unique documentation standard, managed in national terms by the ministry of health since 2016. This means all Portuguese nurses, in primary health care units, use the same documentation pattern, based on the ICNP codification. This is a favorable context to identify, from an epidemiologically approach, the nursing diagnosis of the population. The identification of epidemiological measures like incidence and prevalence of nursing diagnoses, makes possible to look to the health processes of the population and, this way, to understand the more realistic population needs, concerning nursing care. However, at present, the epidemiological surveillance, in its traditional way, focuses on diseases and in risk factors that influence health determinants related to those diseases with the highest prevalence and incidence. Researchers from the 90′s of the twentieth century defended that causal pathways at the societal level and pathogenesis and causality at the molecular level, should be considered in the epidemiology of the future, calling it an eco-epidemiology [5]. Other researchers added to this concept the importance to consider the interactions among individuals and the interplay between individuals and the environment [6]. Frérot and collaborators found evolution of the concepts of epidemiology since the end of 70′s of the twentieth century to nowadays, from a disease-centered epidemiology to a study of the distribution and determinants of health-related states or events (that include diseases as just a dimension to study) [7]. Meanwhile, considering this context as an opportunity to go beyond the epidemiological approach of diseases, we face the challenge to develop what Melo calls a postmodern epidemiology, focused on people and their health-disease phenomena, concerning the nursing point of view [8]. This will provide the opportunity to know the population diagnosis, with the nursing Science approach, considering the metaparadigmatic definition of “person”, related to the intentional processes, to the unintentional processes and to the processes of interaction with the environment, on a macrosystemic point of view [8].

Nurses, in all primary healthcare units of the ACeS, do their records in the same IT software, called “SClínico^®^”. This means it is important that they are all aware of their importance to contribute to epidemiological surveillance of nursing diagnoses. This is the main point that makes us consider the ACeS nurses as a community that needs to be empowered to promote this aim. Hence, from an empowering approach of this community of nurses, considering a process of community partnership and community approach in nursing decision-making [9], this research integrates a main project related with the application of community assessment, intervention and empowerment model (MAIEC) [8,9,10]. In this specific domain MAIEC will be used, as a community health nursing guide model, to promote the epidemiological surveillance of nursing diagnosis. The MAIEC implementation protocol requires the level of empowerment of community to be assessed before the implementation of MAIEC nursing decision matrix [8,9,10,11,12].

The nursing decision matrix of MAIEC has a prescription process, suggesting “community management” as the central focus of nursing diagnosis. “community management”, according to MAIEC concepts, is related to the capacity of the community to lead the solutions to its own problems in an increasing way to community empowerment [8]. To diagnose this focus, MAIEC suggests as diagnostic dimensions the foci “community process”, “community participation” and “community leadership”, all of them codified in ICNP [4]. The diagnose and intervention on these foci, for the nature of the procedures, promotes community empowerment as a process, but also as the result of community health nurse intervention, when this empowerment is assessed before and after the application of MAIEC decision matrix.

To assess community empowerment, Laverack suggest the evaluation of nine domains that structure the power of communities [13,14,15]. These nine domains are:

(a) Community participation (community members and leaders’ involvement);

(b) Problem assessment capacities (the recognition of community capacities to assess its problems);

(c) Local leadership (the existence of leaders related to the solution of community problems);

(d) Organizational structures (the existence of structures, like committees, related to the solution of community problems);

(e) Resource mobilization (the creation and mobilization of resources to promote the solution of community problems)

(f) Links to others (connection to other persons, groups or communities to get resources and solutions)

(g) Ability to "ask why" (community capacity to question the state of the art of its own problems)

(h) Program management (community capacity to develop the managing of a program to solve its problems, independently of outside agents);

(i) Relationship with outside agent (the community dependence on an outside agent to manage and solve its problems).

To evaluate these domains, EARS presents 5 statements for each of them, from whose should be selected one, in a consensual discussion between the community members. The selection of the statement is associated with a score from 1 to 5 that defines the level of empowerment of the community for each specific domain. Melo and collaborators translated to Portuguese and made the cultural validation of EARS to different communities, including ACeS community [16]. The Portuguese version of EARS was called EAvEC [16].

The aim of our study was to assess the level of empowerment of four ACeS to promote the epidemiological surveillance of nursing diagnoses in the domains:

- Community participation;
- Problem assessment capacities;
- Local leadership;
- Organizational structures;
- Resource mobilization;
- Links to others;
- Ability to “ask why”;
- Program management and
- Relationship with outside agent.

## 2. Materials and Methods

This part presents the study design, the participants, the setting and procedures of the study, the data analysis procedures and ethical issues.

### 2.1. Study Design

The study was developed through the technique of focus group, as the first stage of a larger study, which included the application of MAIEC.

This article presents the process and result of this first stage, which through the participatory methodology of focus group allowed the application of EAvEC and the assessment of the level of community empowerment of four ACeS to promote the epidemiological surveillance of nursing diagnoses. Each focus group was developed in the chronological sequence of integration of the ACeS in the study, occurring in September 2017 (one ACES), 2018 (two ACeS) and 2019 (one ACeS).

### 2.2. Participants, Setting and Procedures

The study involved professionals representing all the primary care units of the four ACeS from the north region of Portugal. The selection of the participants had as criteria:

To be a member of the technical council of the primary care unit (the council that works on technical and scientific procedures for each unit) and/or:

- To be a member of the nursing dean of the ACeS and/or
- To be the coordinator of the public health unit.

All participants were invited to participate in this focus group by the executive director of each ACeS and signed an informed consent to be part of the study.

The average duration of each focus group meeting was 75 min and the presentation was the same for all ACeS, following the steps:

(1) Presentation of MAIEC project (structure and specific aims related to the domain of epidemiological surveillance of nursing diagnoses);
(2) Presentation slide-by-slide (using PowerPoint), of the five statements of EAvEC for each of the nine domains, signaling each statement with a letter from A to E;
(3) Then the focus group manager asked, when each domain was presented, that the participants should discuss and consensually select the statement that they considered to characterize the ACeS, concerning to the problematic of epidemiological surveillance of nursing diagnoses;
(4) A redactor from the research team wrote on the printed EAVeC, the statement chosen for each domain, putting relevant aspects of the discussion into observations;
(5) The discussion of each domain ended when most participants selected a statement and the reasons for choosing it. The number of participants who disagreed with the chosen statement was also registered when there were any, as well as the statements they would choose instead. The redactor, in the field of observations, wrote down all this information.

### 2.3. Data Analysis

The results of the focus group, concerning to the EAVeC were transcribed to a Microsoft Excel 2019 database and then presented in a spider web graphic that enabled a visual expression of the level of empowerment of the community for all nine domains.

The observations indicated by the transcriber in the EAvEC were considered to complement the quantitative analysis expressed in the graphic.

The sociodemographic data of the participants were also analyzed using simple descriptive statistical analyses with Excel 2019.

### 2.4. Ethical Issues

The project was submitted to the ethical committee of the Portuguese north regional health administration with a favorable opinion (ethics committee opinions number 51–2017 and 130–2018) and to the ethical committee of local health unit of Matosinhos, also with favorable opinion (ethics committee opinion number 55/CE/JAS/2018).

## 3. Results

In the first part, we present the sociodemographic data of the participants in the focus groups. In Table 1 is presented the distribution of participants, concerning their professional title:

Most part of the participants in the focus group were specialist nurses (63%), followed by general practice nurses (36%) and public health physicians (2%). The physician was the coordinator of one of the four public health units that participated in the study.

From this point on, considering that the community studied in this project was the ACeS nurses’ community, we will present the next data considering just the nurses that participated in the focus groups.

Table 2 presents the distribution of nurses, concerning their specialty:

Specialized nurses were mostly community health nursing specialists (38%), followed by Child Health and Pediatric nursing (20%). In ex aquo there were 13% medical-surgical nursing and rehabilitation nursing specialists. Mental Health and Psychiatric nursing specialists represented 10% of the sample and the lower part of the participants were specialized in maternal health and obstetric nursing (8%).

The distribution of the participants in the different primary care units of the ACeS is presented in Table 3:

Most the participants worked in family health care, representing 74% of the participants. Fifteen percent worked at community care units, 6% worked in public health units, followed by nurses that had their professional exercise in other units, like pneumological diagnosis center or HIV testing and counselling centers (4%) and one nurse that worked at a shared healthcare resource unit, representing 1% of the participants in the focus group.

On Table 4, we present the distribution of nurses concerning their years of experience as a nurse (years of working) and the years of working in the ACeS:

Most part of the nurses had professional experience of between 16 and 20 years (40%), followed by nurses that worked for 10 to 15 years (24%), less than 10 years (16%), from 21 to 25 years (11%), more than 30 years (6%) and 26 to 30 years (3%). Furthermore, most part of the participants worked at the ACeS for 16 to 20 years (48%), followed by nurses that worked in the ACeS for less than 10 years (24%), 10 to 15 years (16%), 21 to 25 years (10%) and 26 to 30 years (3%).

**Figure 2:**
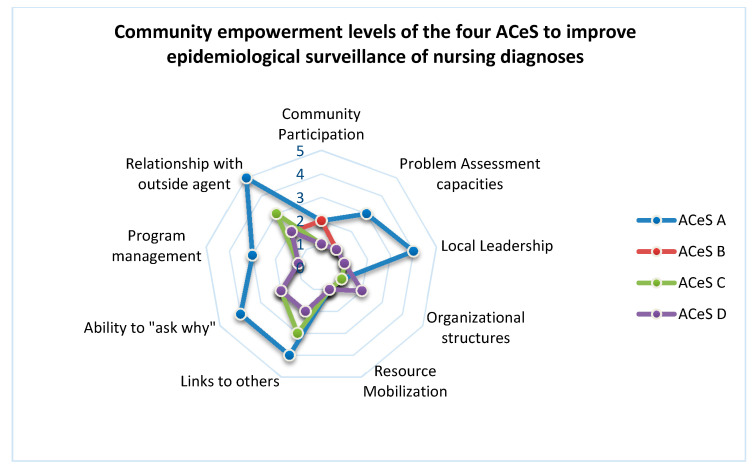
Level of community empowerment of the four ACeS to promote epidemiological surveillance of nursing diagnoses.

Concerning the assessment of the community empowerment of the four ACeS, related to the epidemiological surveillance of nursing diagnosis, we present the results in the Graphic of Figure 2:

Concerning community empowerment level, we found in the four ACeS the lowest level of empowerment in the domain of ”resource mobilization” (level 1 for the four ACeS). There was no resource mobilization (such as human resources, time and task distribution) to promote epidemiological surveillance of nursing diagnosis;

The “organizational structures” and ”community participation” domains varied between level 1 and 2. The biggest part of ACeS presented the lowest level (level 1) of empowerment in these domains (just one ACeS identified level 2 of empowerment). This means that there is not a structure (like a committee or a workgroup) focused on the epidemiological surveillance of nursing diagnoses.

The “community participation” level was also low (just one ACeS chose level 2, with most them choosing level 1). This means there is not any kind of participation of the member of ACeS in activities related to epidemiological surveillance of nursing diagnoses. nurses from the different units do not feel they participate in any kind of activity related do epidemiological surveillance of nursing diagnoses.

In the other domains we found more discrepancies between ACeS. Concerning the “Problem assessment capacities” one of the ACeS considered to be in the level 3, but all other 3 ACeS considered to be in level 1. The better qualified ACeS considered that their professionals had some experience related to nursing documentation in Information Systems and this could be considered as a problem assessment capacity to start first steps in the epidemiological surveillance.

The same ACeS identified higher levels of empowerment related to “relationship with outside agents” (like researchers and political structures). For this ACeS, the research project of our Research Center already represented a “relationship with an outside agent”, although in a starting process. The other 3 ACeS considered this initial relation could not yet be considered a relationship because it would need more time and the definition of formal procedures for the future. The “ability to ask why” (related to awareness for this problematic) and to “Program management” capacities, were also identified in higher levels by the same ACeS, for the same reasons pointed in the domain of “Problem assessment capacities”. The other 3 ACeS, considering that they did not have any process of epidemiological surveillance of nursing diagnosis, indicated for these domains the lower level of the scale. Most the ACeS do not think they are prepared to assess the problem of epidemiological surveillance of nursing diagnoses. They believe they need formal education and training to promote it. They also consider they need to develop formal relationships with outside /external agents (like Universities, Research Centers and the ministry of health).

## 4. Discussion

The promotion of an epidemiology centered in persons, defended by Melo [5] is essential to promote the surveillance of population needs beyond the study of diseases. community and public health nurses are qualified professionals, in primary health care services in Portugal, to develop the epidemiological surveillance of nursing diagnoses in the public health units working with all nurses from all healthcare units from the Portuguese ACeS.

The opportunity to have, in the Portuguese national health service, a central management of parametrization of nurses’ documentation in “primary healthcare IT” it is a great conjuncture to promote the epidemiological surveillance of nursing diagnosis.

The fact that all nurses report their diagnoses and interventions using international classification of nursing practice (ICNP) and all of their documentation is, this way, codified and able to be aggregated in public health data, promotes the possibility to have a macro-analysis of the population needs, concerning nursing diagnoses, using public health measures like incidence and prevalence.

In the matter of Health Planning, this is crucial to help the healthcare units of ACeS to develop realistic and appropriate goals to respond to individuals, families and communities’ needs in order to promote better health status in the population level.

However, the level of empowerment of the assessed ACeS to promote this epidemiological surveillance is still very low and needs an organized intervention focused on ACeS as the target of care of public health nurses, to increase this empowerment levels and promote this new epidemiological approach of populations. As one of the community and public health nursing Specialists competences is the community empowerment [3], there is a great management opportunity for primary health care leaders to ensure that these specialists are allocated to public health units. The participation of the leader of the nursing dean of the ACeS in the focus group was relevant to the understanding of this fact by someone who has the decisions about clinical resources (human and technical).

The results of this study showed an important instrument that nurses are able to use and diagnose the ACeS community empowerment level (EAvEC). In addition, nurses can make the nursing diagnose on this community, concerning to the focus “community management” to promote epidemiological surveillance of nursing diagnoses, using MAIEC decision-making matrix, that we believe will contribute to achieve better community empowerment results, as we are now studying, using EAvEC as vehicle to assess these results).

Adding to these two diagnostic strategies, community and public health nurses can also promote the epidemiological surveillance itself, making the analysis of data produced by nurses in health information systems, with epidemiological measures like incidence and prevalence of nursing diagnosis, because the health information systems already provide that information, if nurses have goals to use it.

Promoting this reality, primary healthcare structures in Portugal will have the opportunity to ensure a process of health planning and program and project management related to the needs of population in nursing care [5]. This way, the population will have the opportunity to access community and public health nursing care, having a response to a more approximated approach of their real health needs.

It is important to highlight that the motivation of all studied ACeS to promote their capacities and ensure the foundations to develop the epidemiological surveillance of nursing diagnoses was a very important finding.

However, it must be referred as a limitation of the study the fact that it was a circumstantial study of these specific ACeS who have their own political and organizational circumstances. The results could be different from other ACeS. But it is important to highlight the fact that Portugal does not yet carry out the epidemiological surveillance of nursing diagnosis and we didn’t find any previous experiences in the assessment of the level of community empowerment, related to this issue, not only in Portugal, but in any other realities. This means that there are no other comparative studies related to the assessment of community empowerment in primary healthcare organizations, concerning to epidemiological surveillance of nursing diagnosis, highlighting the innovation of this project. This fact allows us to have the conviction that the results could be potentially similar to those found with these participants.

## 5. Conclusions

Primary healthcare organizations, like ACeS, can be consider a community that potentially have conditions to be the target of community and public health nurses care, when this care is related to epidemiological surveillance. This requires a change of paradigm in the care of these nursing specialists, using nursing decision models oriented to community as target, like MAIEC, but also promoting the surveillance of population needs in nursing diagnoses. We did not find any evidence of this epidemiological approach being developed in any other primary healthcare system, so this is an innovative process, that as a new process must have a continuous learning and improvement with the evidences that will be produced in the way.

Using the methodological process presented in this study, community and public health nurses will have the chance to identify the level of empowerment of primary healthcare organizations. In addition to this diagnosis nurses can identify the empowerment domains to be improved with an effective community care planning based on community empowerment as a process, but also as a potential result, that is, a health gain sensitive to community and public health nursing care.

EAVeC as an instrument allows comparing the level of community empowerment before and after the intervention of nurses with communities. This is especially important when nurses use conceptual models that allow the objective identification of the concepts, assumptions, postulates and steps on the nursing clinical decision-making process focused on communities. When this clinical decision focuses in community as target of nursing care, we are convinced that MAIEC can be an important nursing model to promote community empowerment and EAVeC a good instrument to assess this impact.

The participation of the leader of the nursing dean of the four ACeS in the focus group was relevant to their understanding of the importance of human and technical resources to be considered as important issues concerning to some of the domains of community empowerment (e.g., organizational structures or community participation).

At the center for interdisciplinary research in health, in Universidade Católica Portuguesa, it is being developed this study related to the epidemiological surveillance of nursing Diagnoses. The results presented in this study are part of the first assessment of community empowerment level of these ACeS. We are currently implementing nursing interventions, using MAIEC clinical decision matrix prescriptions. This process of innovative research is promoting changes in the clinical contexts. For example, related to the result of the inexistence of organizational structures in ACeS focused on epidemiological surveillance on nursing diagnosis, it was promoted the creation of those that were called nursing diagnoses observatories (NDO). There is now an NDO in each of the ACeS that participated in the study, to promote epidemiological surveillance of nursing diagnoses and to improve the empowerment levels of these communities to develop it.

This study is giving contributions:

- To research, offering innovative processes that can be replicated in other communities in order to identify the contribution of community and public health nurses in the increase of community empowerment;
- To clinical practice, giving effective prescriptions to a nursing decision-making promoter of community empowerment as a set of health gains sensitive to community and public health nursing care. The study also contributed to the improvement of the integration of nursing data in an epidemiological approach at the public health units;
- To society, offering populations the right to get access to community and public health nursing care that allow the most effective answers to their real needs concerned to nursing diagnosis.

## Figures and Tables

**Table 1:** Focus group participants profile concerning to professional title.

Professional Title	Fi	%
Nurse (general practice)	40	36
Specialist nurse	23	63
Public health physician	1	2

**Table 2:** Focus group participants profile concerning nursing specialties.

Professional Title	Fi	%
Community health nursing	15	38
Child health and Pediatric nursing	8	20
Medical-surgical nursing	5	13
Rehabilitation nursing	5	13
Mental health and psychiatric nursing	4	10
Maternal health and obstetric nursing	3	8

**Table 3:** Focus group participants profile concerning care unit where they work.

Primary Care Unit	Fi	%
Family health unit	50	74
Community care unit	10	15
Public health unit	4	6
Shared healthcare resource unit	1	1
Other units (pneumological diagnosis center/HIV diagnosis center)	3	4

**Table 4:** Focus group participants profile concerning professional years of working and years of working in the ACeS.

Years of Working	Fi	%	Years of Working in the ACeS	Fi	%
Less than 10 years	10	16	Less than 10 years	15	24
10 to 15 years	15	24	10 to 15 years	10	16
16 to 20 years	25	40	16 to 20 years	30	48
21 to 25 years	7	11	21 to 25 years	6	10
26 to 30 years	2	3	26 to 30 years	2	3
More than 30 years	4	6	More than 30 years	0	0
